# Transcriptome analysis reveals multiple effects of nitrogen accumulation and metabolism in the roots, shoots, and leaves of potato (*Solanum tuberosum* L.)

**DOI:** 10.1186/s12870-022-03652-3

**Published:** 2022-06-09

**Authors:** Heng Guo, Xiuqin Pu, Hao Jia, Yun Zhou, Guangji Ye, Yongzhi Yang, Tiancang Na, Jian Wang

**Affiliations:** Qinghai University/Qinghai Academy of Agriculture and Forestry Sciences/Northwest potato Engineering Research Center of Ministry of Education/Key Laboratory of Qinghai-Tibetan Plateau Biotechnology of Ministry of Education, Xining, 810016 Qinghai China

**Keywords:** Potato (*Solanum tuberosum* L.), RNA-Seq, Nitrogen metabolism, WGCNA, Co-expression

## Abstract

**Background:**

Nitrogen (N) is a major element and fundamental constituent of grain yield. N fertilizer plays an essential role in the roots, shoots, and leaves of crop plants. Here, we obtained two N-sensitive potato cultivars.

**Results:**

The plants were cultivated in the pots using N-deficient and N-sufficient conditions. Crop height, leaf chlorophyll content, dry matter, and N-accumulation significantly decreased under N-deficient conditions. Furthermore, we performed a comprehensive analysis of the phenotype and transcriptome, GO terms, and KEGG pathways. We used WGCNA of co-expressed genes, and 116 differentially expressed hub genes involved in photosynthesis, nitrogen metabolism, and secondary metabolites to generate 23 modules. Among those modules, six *NRT* gene families, four pigment genes, two auxin-related genes, and two energy-related genes were selected for qRT-PCR validation.

**Conclusions:**

Overall, our study demonstrates the co-expressed genes and potential pathways associated with N transport and accumulation in potato cultivars’ roots, shoots, and leaves under N-deficient conditions. Therefore, this study provides new ideas to conduct further research on improving nitrogen use efficiency in potatoes.

**Supplementary Information:**

The online version contains supplementary material available at 10.1186/s12870-022-03652-3.

## Introduction

As a significant cultivated and fertilizer-intensive crop planted worldwide, potato (*Solanum tuberosum* L.) is the third most important food source. Potato serves as an alternative source for energy production [1]. It yields high dry matter and calories per unit space and time. Nevertheless, potato is a good source of energy, proteins, vitamins, and minerals. Potato is consumed by over a billion individuals worldwide, and more than one hundred forty countries presently plant potatoes [2, 3]. However, the cultivation technology and production level of potatoes still needs improvement.

Nitrogen (N) is a constituent of variety of cell elements, like amino acids, proteins, cell walls, membranes, and nucleic acids. In leaves, N deficiency reduces plant growth and development, chemical process, and leaf space and ultimately limits plant productivity [2–5]. There’s a demand to scale back N fertilizer input and increase nitrogen use efficiency (NUE). This could be achieved by understanding the connection between N nutrition and the chemical action rate within the leaf [6–8]. Additionally, the best levels of N uptake and utilization efficiency were obtained for nitrate-fed plants. This resulted in the highest dry biomass, N content, leaf chlorophyll, gas exchange, and root growth [9, 10]. Previous experiments showed necessary root traits in each high- and low-N environments. For example, in high-N environments, increasing root biomass [11] and root length density [12, 13] were shown to be related to larger N uptake and yield. Studies in N-deficient environments report that raised ‘early vigor’ [14], raised root: shoot ratios [15], and lower specific root lengths [16] are related to higher productivity. Low-N stress will increase shoot-to-root growth regulator transport, which enhances root elongation. This happens via auxin-dependent acid growth and therefore also the auxin-regulated target of the rapamycin (TOR) pathway in plants [17, 18]. N fertilizer and NUE play essential roles in crop plants’ roots (R), shoots (S), and leaves (L). Numerous genes, like nitrate reductase transporters (*NRTs*), nitrate reductase (*NR*), glutamine synthetase (*GS*), glutamate dehydrogenase (*GDH*), and nitrite reductase (*NIR*), are known to be related to N absorption and utilization [19, 20]. Therefore, understanding N responsiveness and organic phenomenon in potato is necessary for high-NUE potato selection breeding for the best potato genotype has each high genetic NUE and high N reactivity.

Transcriptome databases offer a valuable resource for genetic and genomic studies in plant species. Genes concerned in N accumulation and metabolism, photosynthesis, and hormone biosynthesis are identified via transcriptome sequencing (RNA-Seq) and analysis [2–6, 21]. In the present study, we identified multiple genes, gene modules and metabolic pathways in our native potato cultivars by using RNA-Seq and gene co-expression analysis. We first obtained root, shoot, and leaf tissues from N-deficient and N-sufficient treated potato plants and then identified genetically and genomically expressed variation via informatics analyses. Finally, we examined the relationship between multiple stress treatments and N metabolism to provide new information to improve NUE in crop plants.

## Materials and methods

### Plant materials and experimental treatments

Two potato cultivars, Q9 (Qingshu9, with a growth period of 125 days) and 65 (Xiazai65, with a growth period of 125 days), were planted in pots in a greenhouse (conditions: 26 ± 2 °C, 60% relative humidity, 14 h light/10 h dark) at Qinghai Academy of Agriculture and Forestry Sciences. Q9 and 65 are provided by Qinghai Academy of Agriculture and Forestry Sciences. The two potato cultivars were divided into the N0 (N deficiency) and the N1 (N sufficiency) groups for the N treatment. Plants in the N0 group were treated with 0 g N (Urea, N:46%), 13 g P (P_2_O_5_:12%), and 4.9 g K (K_2_O:40%); Plants in the N1 group were treated with 3.4 g N (Urea, N:46%), 13 g P (P_2_O_5_:12%), and 4.9 g K (K_2_O:40%). The amount of compound fertilizer was converted and mixed according to the whole pots. After a growth stage of 50 days, new, fresh roots, shoots, and leaves of Q9 and 65 plants were collected separately for transcriptome sequencing analysis. Equal amounts of leaves were selected for physiological and biochemical parameter measurements. For RT-qPCR validation, fresh tissues from Q9 and 65 plants were sampled in tubes, frozen in liquid nitrogen, and then stored at − 80 °C until analysis.

### Physiological and biochemical parameter assays

Twelve plants in each group were selected, four were pooled as a repeat, and three biological replicates were set. The plant heights of each group of Q9 and 65 plants were measured using a meter stick (Qinghai, China). Moreover, the chlorophyll contents of potato leaves were measured using a SPECORD 200 spectrophotometer (Analytik Jena, Germany) according to a previously reported method [22, 23]. Leaf extractions containing chloroplast pigments were measured for specific light absorption at 665 and 649 nm. The chlorophyll content was calculated as follows: Chl = 13.95 × A_665–6.88 × A_649 + 24.96 × A_649–7.32 × A_665. The dry matter of the roots, shoots, and leaves was measured by using the following method: the divided tissues were heated in an oven (105 °C, 30 min) and then dried to constant weight (70 °C, 8 h); the dry matter was weighed on an electronic balance (METTLER TOLEDO, Shanghai, China). Statistical analysis of plant morphological data was conducted using SPSS 19.0 (IBM, Chicago, IL, USA). N accumulation was also determined using an ultraviolet spectrophotometer and a methylthymol colorimetric method. Statistical results were obtained by one-way analysis of variance (ANOVA) followed by Tukey’s test to evaluate significant treatment effects.

### Potato RNA isolation and detection

The roots, shoots, and leaves of Q9 and 65 potato plants were collected separately. Each leaf sample was obtained by sampling the completely expanded fourth leaf. Moreover, we selected the stem tissue between the fourth leaf and the fifth leaf. In addition, we washed and dried the water after sampling the root tissue of the plants. Then, 36 samples (two cultivars, N0 and N1 treatment, three tissues) with three biological replicates were prepared for RNA extraction (TRIzol-A+ reagent, TIANGEN BIOTECH, Beijing) followed by treatment with RNase-free DNase I (TaKaRa). RNA quantity was measured with a Nanodrop and Qubit 2.0 Fluorometer (Life Technologies, CA, USA). RNA quality was evaluated with an Agilent Bioanalyzer Model 2100 (Agilent Technologies, Palo Alto, CA). Samples with an RNA integrity number (RIN) value greater than 6.6 were deemed acceptable according to the Illumina transcriptome sequencing protocol of the Beijing Allwegene Technology Company (Beijing, China) [24].

### Library construction and transcriptome sequencing

In total, 36 cDNA libraries were constructed by using the NEBNext® UltraTM RNA Library Prep Kit for Illumina® (#E7530L, NEB, USA). Following the protocol, poly (A) mRNA of the Q9 and 65 potato groups was enriched using oligo (dT) magnetic beads and then broken into small pieces using fragmentation buffer. These mRNA fragments were used as templates for cDNA synthesis. First-strand cDNA was synthesized using reverse transcriptase and random primers. This was followed by second-strand cDNA synthesis using DNA Polymerase I and RNase H. The well-constructed cDNA libraries were sequenced on an Illumina HiSeq 4000 (Allwegene, Beijing, China) after processing by a QIAquick PCR kit, end repair, and sequence-adapter joining. The raw reads in fastq format were first processed through in-house Perl scripts. In this step, clean reads were obtained by removing reads containing adapter, poly-N reads, and low-quality reads from the raw reads. At the same time, the Q30 and GC content of clean data were calculated. Mapping of the clean reads was performed using STAR (v2.5.2b) [25] according to the reference genome from Ensembl_plant_release 47 *Solanum_tuberosum*. SolTub_3.0 (ftp://ftp.ensemblgenomes.org:21/pub/plants/release-47/fasta/solanum_tuberosum/) [26].

### DEG identification and enrichment analysis

Gene expression levels were estimated by fragments per kilobase of transcript per million mapped reads (FPKM) using HTSeq [27]. FPKM values were calculated using RSEM [28]. Differentially expressed genes were identified using the DEseq R package [29]. DEseq provided statistical routines for determining differential expression using a model based on the negative binomial distribution. The resulting *P*-values were adjusted using Benjamini and Hochberg’s approach for controlling the false discovery rate (FDR). An adjusted *P*-value ≤0.05 and |log2 fold changes| ≥ 1 were used as the thresholds of differential expression. In addition, DEGs were annotated with ShinyGO (v0.61, http://bioinformatics.sdstate.edu/go/) and KOBAS (v2.0, http://kobas.cbi.pku.edu.cn/) assignments to obtain significantly enriched GO (Gene Ontology) terms and KEGG (Kyoto Encyclopedia of Genes and Genomes) pathways [30–32]. GO terms with FDR corrected at a *P*-value ≤0.05 were regarded as significantly enriched [33], and pathways with FDR corrected at a *P*-value ≤0.05 were considered significantly enriched [34].

### Weighted gene co-expression network analysis (WGCNA)

The gene co-expression regulatory network was constructed using the WGCNA (v1.29) package in R. The detailed analysis methods were based on a previous study [35–37]. A total of 20,594 genes with an average FPKM> 1 from three replicates were selected for the WGCNA network analysis. The appropriate power value in this study was determined to be eight. The modules were obtained by the automatic network construction function with default parameters in the WGCNA software package. The correlation between the modules and traits was calculated by the Pearson method using the blockwise module function. The top ten genes with maximum intramodular connectivity were considered “highly connected genes” (hub gene).

### Quantitative real-time PCR (qRT-PCR) validation

qRT-PCR analysis was employed to verify the DEG results using Bio-Rad CFX Manager (Bio-Rad, CA, USA) with SsoFastTM EvaGreen Supermix (Bio-Rad). Primers for specific N metabolism, photosynthesis, and chlorophyll genes were designed using Beacon Designer 7 (Bio-Rad, USA). Gene-specific primer sequences for qRT-PCR are listed in Table S1. qRT-PCR assays were performed in triplicate (technical repeats) with three independent biological replicates, with StActin as the internal standard based on the following method [38]: 1 μg of total RNA from the same batch of RNA for high-throughput RNA-Seq was used for first-strand cDNA synthesis using iScript (Cat#1708891, BioRad, Hercules, CA, USA) according to the supplier’s protocol. PCR was conducted in a total volume of 20 μL with 2 μL of cDNA template, 400 nM forward primer, 400 nM reverse primer, and 14 μL of SsoFast EvaGreen Supermix (Cat. #1725200, Bio-Rad, Hercules, CA, USA). Quantification was determined by BioRad CFX manager software (V3.1). Quantitative verification was performed by a relative quantitative method (2^−ΔΔCT^) [38].

### Data availability

The RNA-Seq data of 36 potato samples have been uploaded to the NCBI SRA database, and the Bioproject accession: PRJNA741081(https://www.ncbi.nlm.nih.gov/bioproject/PRJNA741081/).

## Results

### N treatment affects the morphology of potato plants

**Fig. 1:**
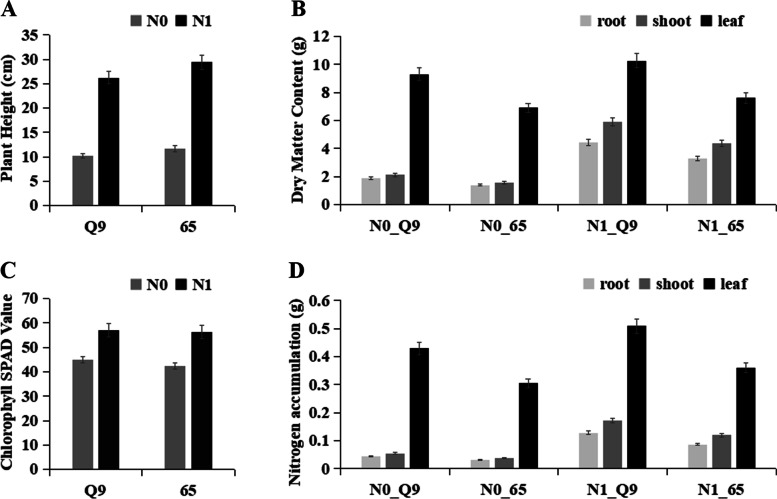
Physiological and biochemical parameters in two potato cultivars. **A** The plant height of Q9 and 65 potato cultivars under N0 and N1 conditions. **B** The dry matter weight of the potato root (R), shoot (S), and leaf (L). **C** The chlorophyll contents in Q9 and 65 potato leaves. **D** The nitrogen accumulation content in the potato R, S, and L

Potato cultivars Q9 and 65 under the N0 and N1 treatments were observed for plant height; leaf chlorophyll content; leaf, shoot, and root dry weight; and N accumulation contents (Fig. 1). Potato plants were highly sensitive to N levels. Both cultivars in the N0 group showed lower plant heights than those in the N1 group (Fig. 1A). The change patterns for chlorophyll contents or SPAD remained consistent with that of plant height. N sufficiency improved the chlorophyll accumulation content in potato leaves in Q9 and 65 (Fig. 1C). The dry matter and nitrogen accumulation showed that the contents in leaves were better than those in roots and shoots. Furthermore, Q9 had higher dry matter and N accumulation levels than 65 (Fig. 1B and D), suggesting that Q9 had a higher NUE than 65. The soil N absorption and transport moved from the roots to the shoots and accumulated in the leaves.

### RNA-Seq results and analyses

To verify the mechanism of N metabolism in Q9 and 65 under the N0 and N1 conditions, we used transcriptome analysis to identify genes. The quality and quantity of the 36 RNA samples were high, with an RNA integrity number (RIN) greater than 6.6 (Table S2). In addition, 36 cDNA libraries from Q9 and 65 plants produced more than 37,000,000 paired-end clean reads, and the Q30 values were higher than 92%. The total reads were mapped to the reference genome from Ensembl_plant_release 47 *Solanum_tuberosum*. SolTub_3.0. More than 92% of the clean reads from each sample could be mapped, and the GC content was normal at the 42% level (Table S3). These results suggested that the sequencing and genetic data were high quality and provided a basis for the following analyses.

**Fig. 2:**
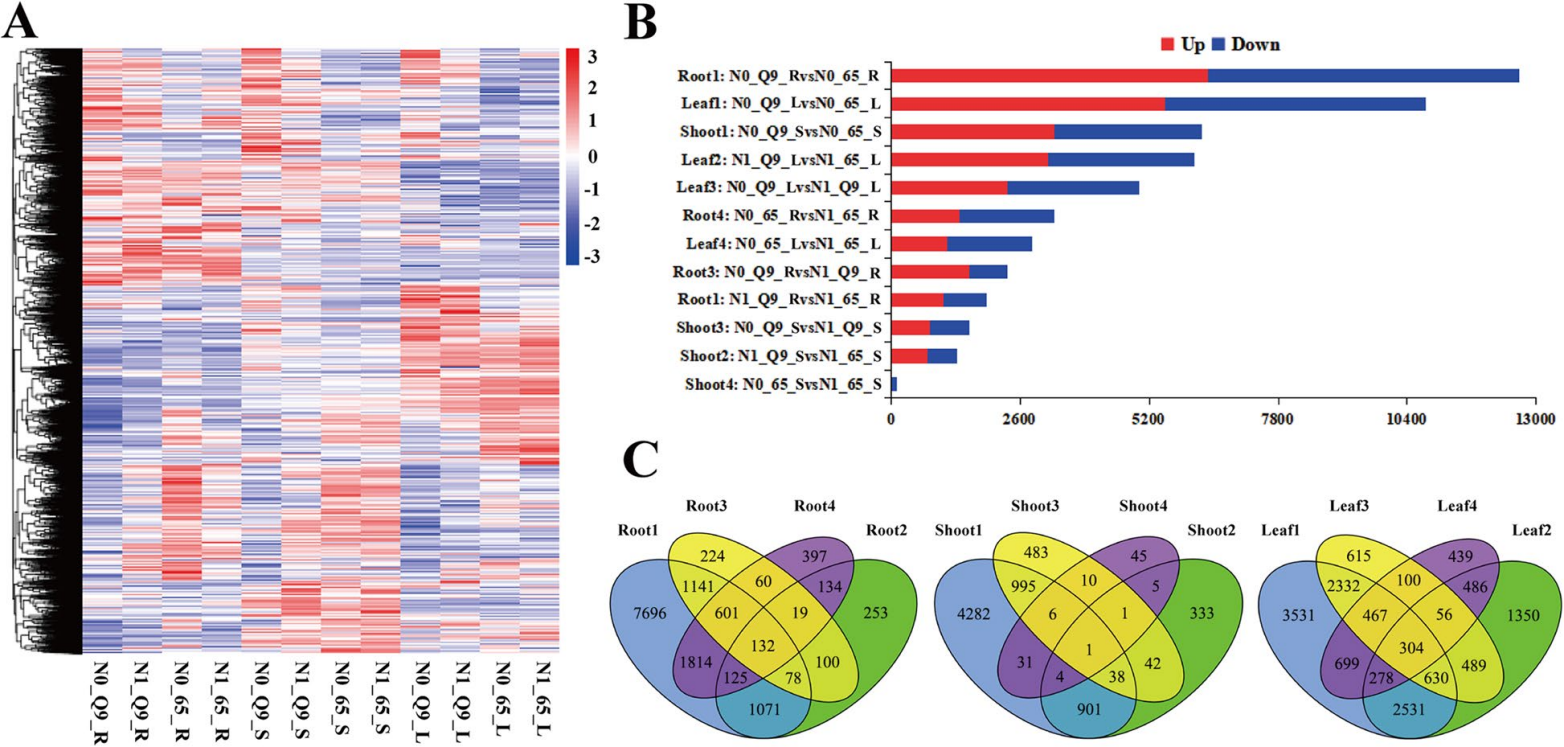
Differentially expressed genes (DEGs) between two potato cultivars’ root (R), shoot (S), and leaf (L) under N0 and N1 treatment. **A** The heat-map of all DEGs in 12 comparisons. **B** The up-and down-regulated DEGs in the 12 comparisons of R, S, and L. **C** The divided Venn analyses of R, S, and L

For DEG analysis, the DEG heat map shows that roots (abbreviated “R”), shoots (abbreviated “S”), and leaves (abbreviated “L”) were clustered into three groups: group 1: N0_Q9_R, N1_Q9_R, N0_65_R, and N1_65_R; group 2: N0_Q9_S, N1_Q9_S, N0_65_S, and N1_65_S; and group 3: N0_Q9_L, N1_Q9_L, N0_65_L, and N1_65_L. The gene expression patterns in each group were similar. However, the gene cluster profiles were different in different plant tissues. The whole comparison groups of R, S, and L are shown in Fig. 2A and Table S4. For the whole comparison groups, Root 1–4 represent N0_Q9_R vs N0_65_R, N1_Q9_R vs N1_65_R, N0_Q9_R vs N1_Q9_R, and N0_65_R vs N1_65_R; Shoot 1–4 represent N0_Q9_S vs N0_65_S, N1_Q9_S vs N1_65_S, N0_Q9_S vs N1_Q9_S, and N0_65_S vs N1_65_S; and Leaf 1–4 represent N0_Q9_L vs N0_65_L, N1_Q9_L vs N1_65_L, N0_Q9_L vs N1_Q9_L, and N0_65_L vs N1_65_L. The N0 nitrogen treatment caused most genes to be differentially expressed between Q9 and 65 in the roots, leaves, and shoots. This was followed by the N1 nitrogen treatment in leaves between Q9 and 65, as well as other comparison groups (Fig. 2B). The Venn analysis of the roots, shoots, and leaves of four groups showed many overlapping and specific DEGs. The leaf comparison groups shared the most DEGs, followed by the root and shoot comparison groups, which generated the fewest DEGs (Fig. 2C and Table S5). These results suggest that Q9 and 65 were more sensitive to N deficiency, and the leaves were most vulnerable to changes in N nutrition.

### DEG classification and GO functional annotation

**Fig. 3:**
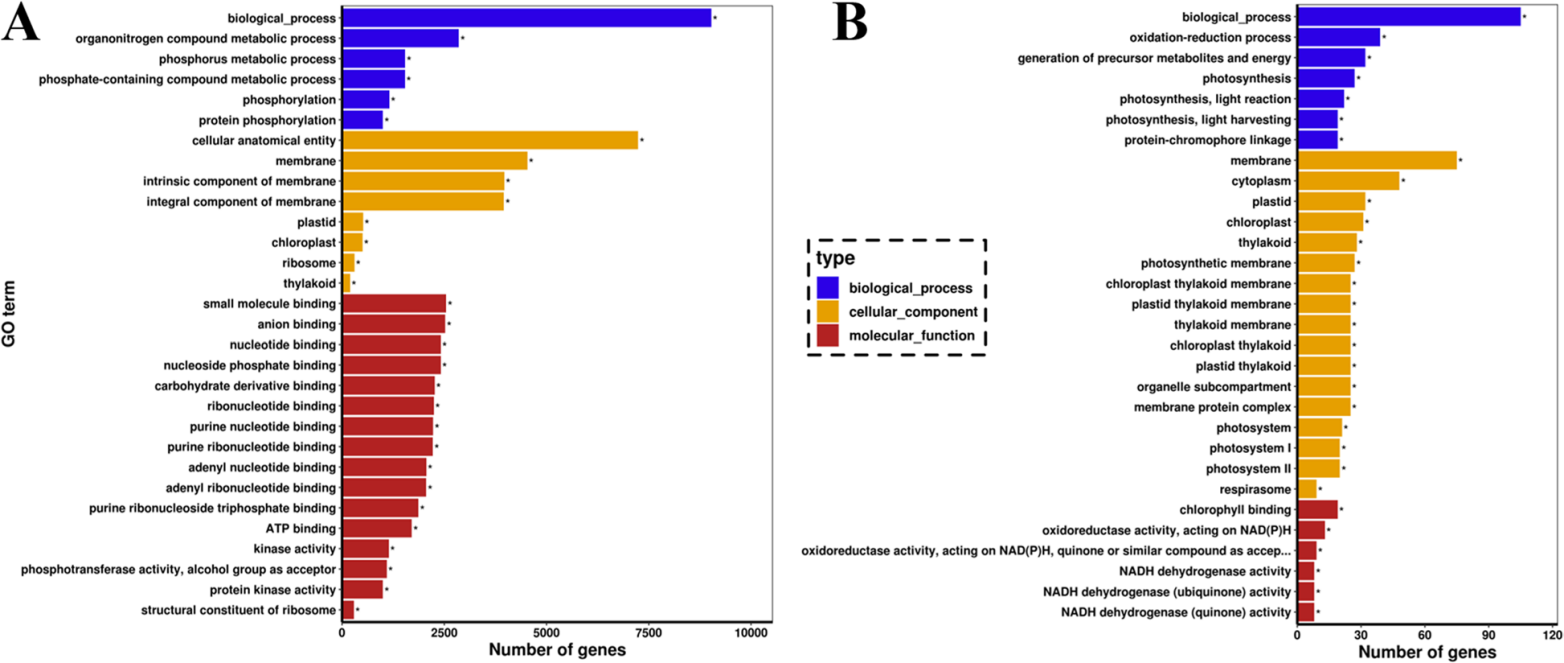
The GO enrichment of DEGs and hub-genes in the root (R), shoot (S), and leaf (L) of N9 and 65 potatoes under N0 and N1 treatment. **A** The top30 enriched GO terms of all the DEGs. **B** The hub-genes top30 GO enrichment result. * represent a corrected *P*-value<0.05

To classify the DEG functions, we used the GO database to divide the DEGs into three categories (Blue: biological process, Yellow: cellular component, and Red: molecular function). Among these GO terms, “organonitrogen compound metabolic process,” “phosphate and containing compound metabolic process,” and “phosphorylation” from the biological process category were mainly enriched. Moreover, “cellular,” “membrane,” and “plastid containing chloroplast and thylakoid” were enriched in the cellular component category, and in the molecular function category, “binding” was the major GO term (Fig. 3A and Table S6). The selected 116 hub genes (Table S7) mainly were enriched in “photosynthesis (including PS I and PS II),” “membrane,” “chloroplast,” and “chlorophyll-binding” GO terms (Fig. 3B and Table S6). These results show that N deficiency and sufficiency affect the photosynthetic process, N transport, and metabolic process. Roots also adapt to the growth process of different light signals through photomorphogenesis by sensing light signals from the ground. Light signals can help roots better adapt to the soil environment [39, 40].

### Significant KEGG pathways of DEGs in roots, shoots, and leaves

Total DEGs in roots, shoots, and leaves under the N0 and N1 treatments were examined using the KEGG database. The results contained multiple significant pathways, including “Biosynthesis of secondary metabolites,” “Photosynthesis-antenna proteins,” “Plant hormone signal transduction,” and “Nicotinate and nicotinamide metabolism.” DEGs in metabolic pathways, such as secondary metabolites and amino acids, were the most enriched in the roots. The most enriched shoots were ABC transporters, secondary metabolites, flavonoids, monoterpenoids, starch and sucrose, and vitamin biosynthesis and metabolism. Carotenoid biosynthesis, other pigments, and photosynthesis were significantly enriched in the leaves. In addition, several DEGs were enriched, as shown in Table 1. These results suggested that differential expression of functional genes might affect multiple metabolic pathways in different tissues of potato cultivars.

**Table 1:** The most enrichment KEGG pathways of root (R), shoot (S), and leaf (L) DEGs

KEGG term	KO_ID	Root_DEGs	Shoot_DEGs	Leaf_DEGs	*p*_value
ABC transporters	sot02010	0.192	0.040	0.199	<0.001
alpha-Linolenic acid metabolism	sot00592	0.031	0.031	0.015	0.001 ~ 0.005
Arginine and proline metabolism	sot00330	0.072	0.182	0.033	0.005 ~ 0.01
beta-Alanine metabolism	sot00410	0.085	0.020	0.109	0.01 ~ 0.05
Biosynthesis of secondary metabolites	sot01110	0.003	0.001	0.004	0.05 ~ 0.1
Butanoate metabolism	sot00650	0.029	0.203	0.253	>0.1
Carbon fixation in photosynthetic organisms	sot00710	0.067	0.441	0.015	
Carotenoid biosynthesis	sot00906	0.014	0.038	0.001	
Cyanoamino acid metabolism	sot00460	0.096	0.142	0.037	
Ether lipid metabolism	sot00565	0.046	0.053	0.038	
Flavone and flavonol biosynthesis	sot00944	0.054	0.030	0.361	
Flavonoid biosynthesis	sot00941	0.008	0.001	0.501	
Glutathione metabolism	sot00480	4.02E-05	0.158	0.184	
Glycerolipid metabolism	sot00561	0.025	0.091	0.004	
Glyoxylate and dicarboxylate metabolism	sot00630	0.142	0.361	0.043	
Histidine metabolism	sot00340	0.078	0.220	0.015	
Homologous recombination	sot03440	0.027	0.008	0.052	
Inositol phosphate metabolism	sot00562	0.191	0.266	0.020	
Limonene and pinene degradation	sot00903	0.113	0.065	0.025	
Metabolic pathways	sot01100	0.001	0.059	0.084	
Monoterpenoid biosynthesis	sot00902	0.005	0.005	0.182	
Nitrogen metabolism	sot00910	0.226	0.752	0.236	
Nicotinate and nicotinamide metabolism	sot00760	0.076	0.011	0.044	
Phenylalanine metabolism	sot00360	0.058	0.021	0.028	
Phenylalanine, tyrosine and tryptophan biosynthesis	sot00400	0.046	0.573	0.383	
Phenylpropanoid biosynthesis	sot00940	0.092	0.011	0.023	
Photosynthesis	sot00195	9.59E-05	0.007	0.366	
Photosynthesis - antenna proteins	sot00196	1.93E-05	1.34E-11	2.65E-07	
Plant hormone signal transduction	sot04075	2.11E-07	0.004	0.030	
Porphyrin and chlorophyll metabolism	sot00860	0.062	0.053	0.029	
Protein processing in endoplasmic reticulum	sot04141	0.038	0.297	0.672	
Ribosome	sot03010	0.001	4.03E-12	0.002	
Sesquiterpenoid and triterpenoid biosynthesis	sot00909	0.004	0.015	0.077	
Starch and sucrose metabolism	sot00500	0.129	0.007	0.045	
Tryptophan metabolism	sot00380	0.044	0.029	0.001	
Tyrosine metabolism	sot00350	0.244	0.010	0.246	
Valine, leucine and isoleucine biosynthesis	sot00290	0.026	0.312	0.544	
Vitamin B6 metabolism	sot00750	0.035	0.009	0.081	

### Identification of a weighted gene co-expression network

**Fig. 4:**
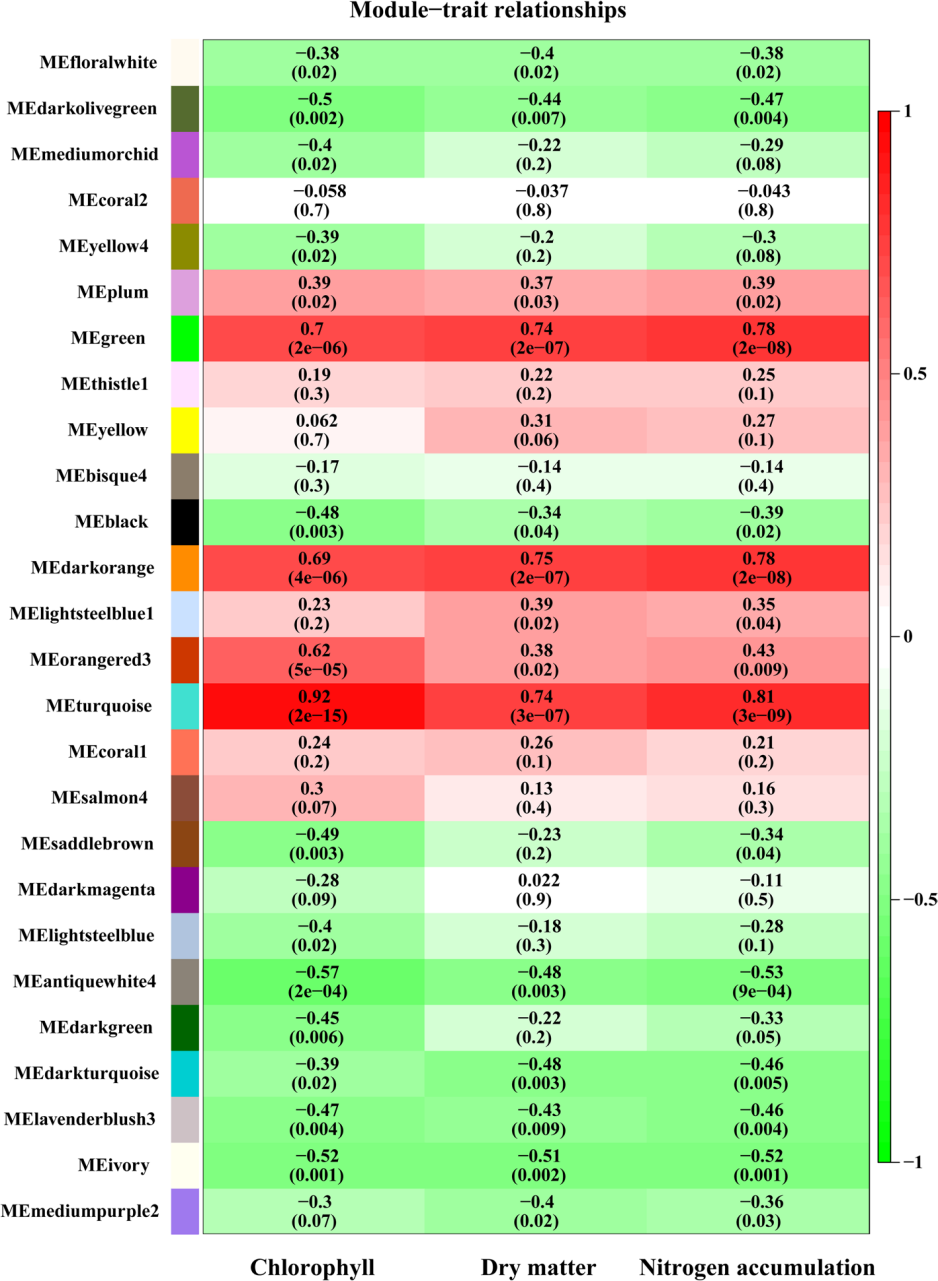
Weighted gene co-expression network analysis (WGCNA) of gene expressions and the related traits

WGCNA was used to obtain candidate key genes or hub genes associated with the related phenotypic traits, including chlorophyll content, dry matter weight, and nitrogen accumulation of roots, shoots, and leaves. After removing the genes with low FPKM levels, 20,594 genes were retained for further analysis. A total of 23 modules (labeled in different colors) were obtained, and three modules (MEturquoise, MEdarkorange, MEgreen) were significantly related to the various phenotypical traits noted above (Fig. 4). The relationships of the 23 modules are shown in Fig. S1. The MEturquoise module had higher correlation values of 0.92, 0.74, and 0.81 for chlorophyll, dry matter, and N accumulation. MEdarkorange showed 0.69, 0.75, and 0.78, and MEgreen showed 0.7, 0.74, and 0.78 for those traits. The eigengenes in the three modules were conserved and significant for further mining and analysis.

### Hub genes involved in N-related and N-affected metabolic pathways

**Fig. 5:**
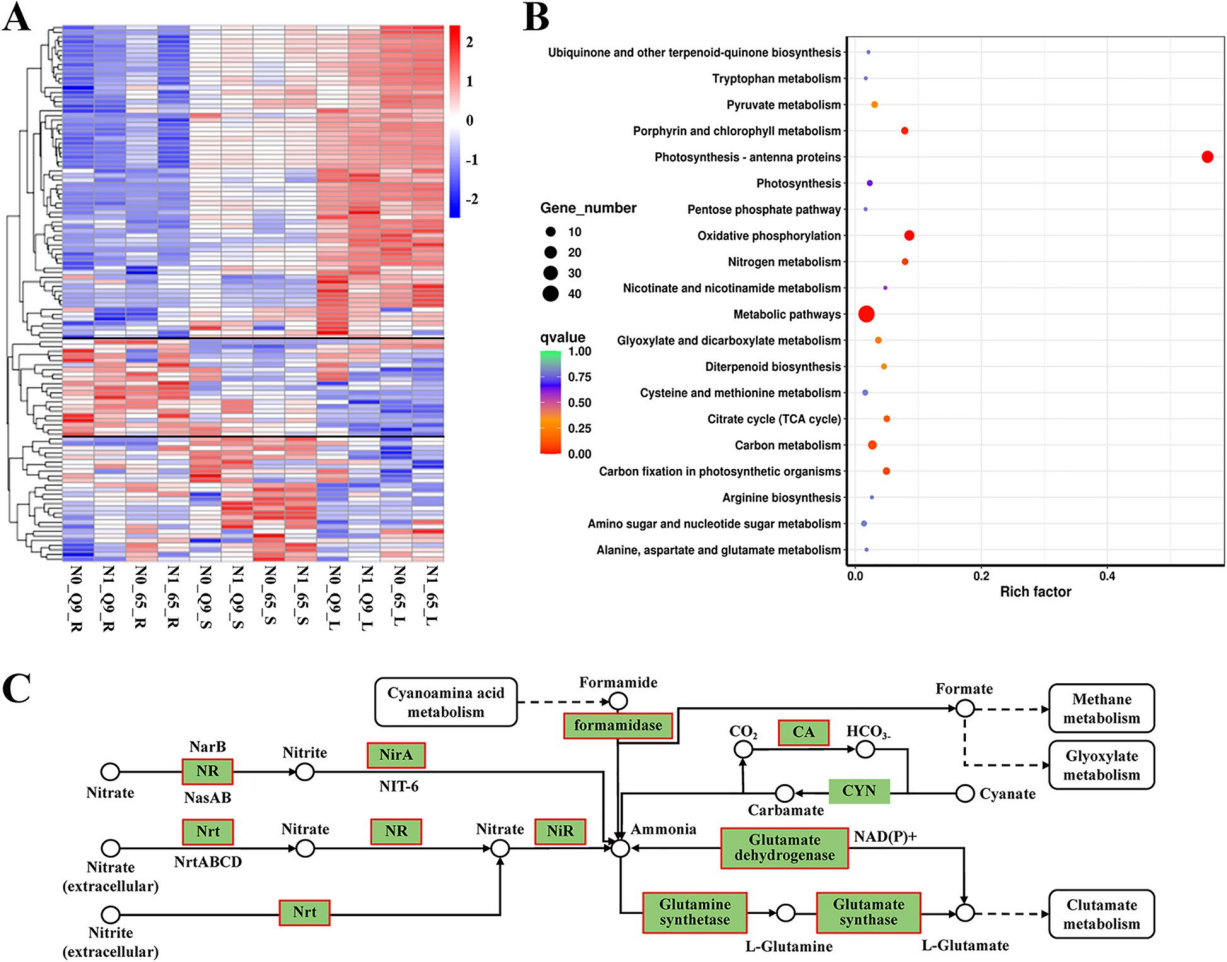
The selected 116 DEGs are involved in N absorb, transport, and biosynthesis metabolism. **A** Heat-map of 116 hub-genes. **B** The top20 enriched KEGG pathways of 116 DEGs. **C** The nitrogen metabolism of potato cultivars and DEGs involved in the pathway (www.kegg.jp/kegg/kegg1.html)

Furthermore, 116 hub genes and DEGs from the three modules in the above WGCNA results were analyzed. The expression heat map shows three cluster profiles. Among these, profile 1 DEGs are gradually upregulated from the roots to the shoots to the leaves. Profile 2 DEGs are downregulated from the roots to the leaves. Profile 3 DEGs are upregulated in the shoots and slightly downregulated in the roots and leaves (Fig. 5A, Table S7). Then, the KEGG pathway enrichment analysis of 116 DEGs showed that 19 were enriched in photosynthesis-antenna proteins (Fig. 5B and S2), 46 in metabolic pathways, and 8 in nitrogen metabolism (Fig. 5B and C). The metabolic pathways including N-glycan, starch, lipid acid, amino acid, pigments, and vitamins. The 8 DEGs *NR, NRT, NIR, NIRA, GS, GDH, CA,* and *formamidase* play important roles in methane metabolism, glyoxylate metabolism, and glutamate metabolism, which are associated with N metabolism. These DEGs and their functions are essential to potato NUE and trait variations.

### Verification of selected DEGs via qRT-PCR

**Fig. 6:**
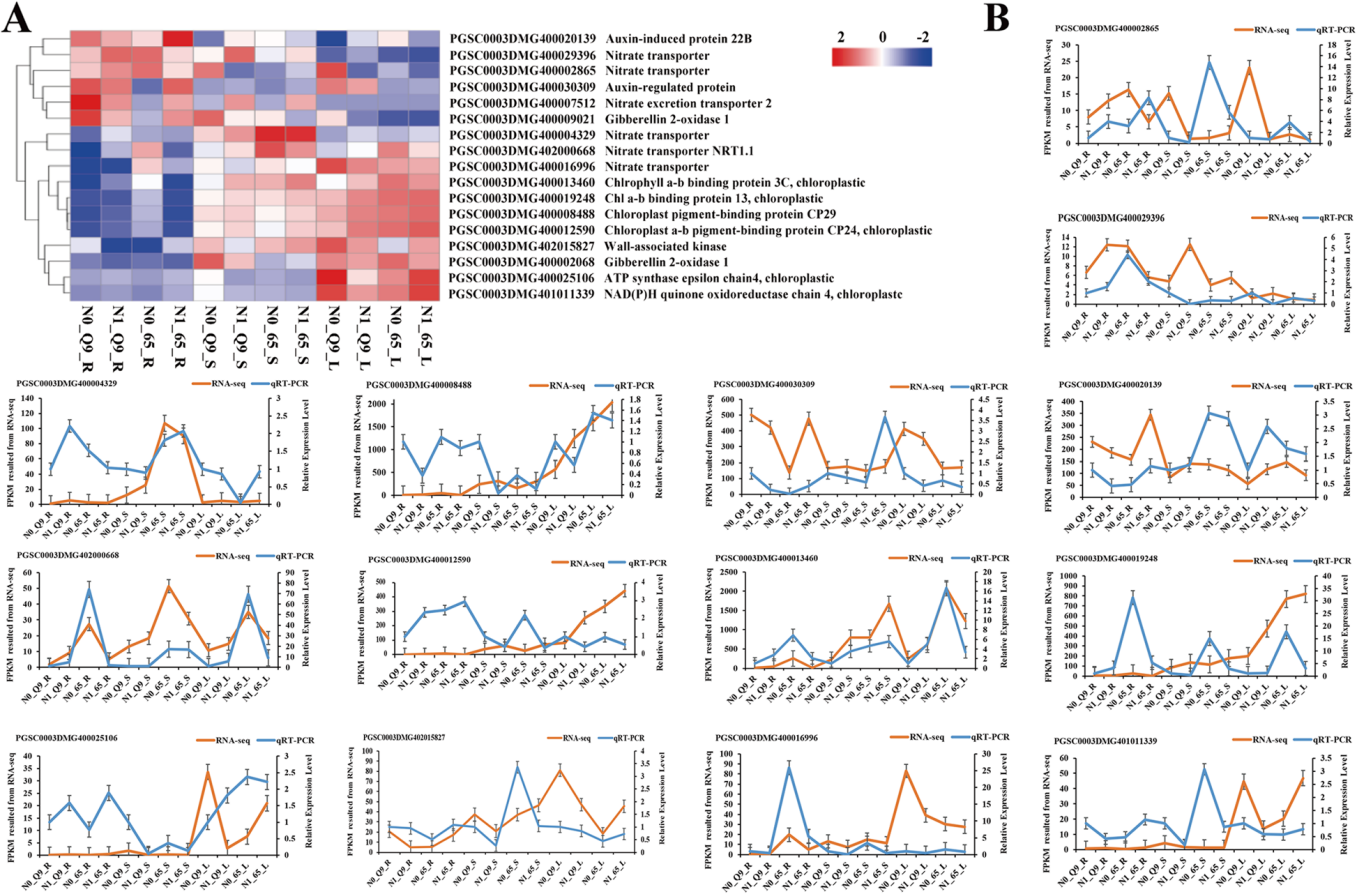
The qRT-PCR validation of fourteen DEGs involved in potato root (R), shoot (S), and leaf (L) under N0 and N1 conditions. **A** The heat-map of selected validation DEGs. **B** The fourteen DEGs expression pattern in N9 and 65 according to the qRT-PCR method

qRT-PCR was performed to validate the transcriptome analysis. Fourteen key DEGs participating in photosynthetic, nitrogen, chlorophyll, and hormone metabolism were selected (Fig. 6). The expression levels of 14 DEGs based on RNA-Seq analysis are shown in Fig. 6A. Four DEGs (PGSC0003DMG400002865: *NRT*, PGSC0003DMG400020139: auxin-induced, PGSC0003DMG400029396: *NRT*, and PGSC0003DMG400030309: auxin-regulated) were upregulated in roots, and 2 DEGs (PGSC0003DMG400004329: *NRT* and PGSC0003DMG402000668: *NRT*1.1) were upregulated in shoots. The other 8 DEGs (PGSC0003DMG400008488: chloroplast pigment-binding, PGSC0003DMG400012590 and PGSC0003DMG400013460: chlorophyll a-b binding, PGSC0003DMG400016996: *NRT*, PGSC0003DMG400019248: chlorophyll a-b binding, PGSC0003DMG400025106: ATP synthase, PGSC0003DMG402015827: wall-associated kinase, and PGSC0003DMG401011339: NADPH) were all upregulated in leaves. The expression data and regulation patterns were similar compared with the corresponding values from the qRT-PCR analyses (Fig. 6B). Five *NRT*s, three pigment genes, two auxin-related genes, and two energy-related genes were consistent with those obtained by qRT-PCR. Generally, the results of selected target DEGs between RNA-Seq and qPCR show that the data are consistent for relevant genes and pathways.

## Discussion

This study bred two new cultivated potato varieties, Q9 and 65, with high-yielding and high-resistance properties in Qinghai, China. We mainly focused on the phenotypic traits and correlated genetic information in the roots, shoots, and leaves of Q9 and 65 under N deficiency and sufficiency. RNA-Seq analysis is an efficient method to study genome-wide changes in gene transcription and to screen existing gene resources in response to different N concentrations [2–5]. Previous studies showed N-responsive genes using two approaches: one involves reducing and/or depletion of N in the growth media, work low-N stress (N starvation). The opposite is achieved by resupplying traditional N to seedlings mature in media with no or very little N, targeted on NUE. Key genes functioning in plant survival were concerned in response to N starvation, including those involved in the overall stress response, chlorophyll synthesis, and N assimilation.

Furthermore, N absorption and assimilation genes were upregulated throughout N supplementation [21, 33, 41–43]. To enhance NUE, it’s essential to grasp the plant response to N treatments, particularly to N limitation at each the physiological and transcriptomic levels. During this study, the candidate genes selected were supported by the previous studies, and can have a potential role in rising NUE [1, 3, 44].

Multiple responsive mechanisms to N treatment are known in crop plants. Among these, photosynthesis, PS I and PS II, N metabolism, transcription factors, and hormone signaling, were associated with mechanisms of N treatment and tolerance [4, 45–49]. However, our study integrated all hub genes related to traits that occurred in the roots, shoots, and leaves of potatoes (Figs. 2, 4, 5). We first analyzed the potato plant heights, chlorophyll contents, dry matter levels, and N accumulation contents (Fig. 1). Both potato cultivars are sensitive to N deficiency conditions. This ends up in a smaller plant height, lower leaf chlorophyll contents, and fewer dry matter weight and N accumulation, particularly in roots and shoots. These results are in accordance with those of a previous study [2–8]. In plants, photosynthesis plays a decisive role in carbon fixation and biomass accumulation. In higher plants, the sunshine reaction of photosynthesis is accomplished by the two photosystems PS I and PS II. These two photosynthesis act in series through the photosynthetic energy transport chain. They’re concerned in the light-dependent reactions of carbon fixation [50]. In the present study, nineteen genes involved in the photosynthesis pathway were downregulated below N deficiency (Table 1, Fig. 5B and S2). All nineteen were enriched in the light-harvesting chlorophyll protein complex (LHC), such as Lhca/b1, Lhca/b 2, Lhca/b 3, Lhca/b 4, and Lhcb6, which bind to PS I and PS II. The results suggest that N nutrition and NUE enhance the photosynthetic pathways. Besides, those genes also are enriched in GO terms “phosphate and containing compound metabolic process,” “photosynthesis,” “membrane,” and “binding” (Fig. 3). All results show the interconnected relationship between photosynthesis and N metabolism.

Previous studies demonstrated potential roles of those N metabolism-associated genes, especially transporters under N stress tolerance in potato [2, 51, 52]. *NRTs* are responsible for the absorption of nitrate from soil and translocation among completely different components of plants. They deliver nitrate wherever required and take measures in addressing adverse environmental conditions [53].

Moreover, as per the previous report, the *NRT* family was found to be concerned in root growth, flowering time, and transcriptional regulation of multiple physiological processes, hormonal and nitrate signaling [54–56]. The upregulated DEGs included members of *NIR* and *NRT* gene families, which increased crop nutrient uptake [57]. We find several *NRT* gene families (Figs. 5C and 6, Table S7). Interestingly, the expression of *NRT* gene family members in N-deficient potato groups was upregulated in root and shoot. We also found that the aminoalkanoic acid, organic compound transporters, and basic principle transporters play key roles in N uptake and transformation from potato root to shoot, and lastly, to leaf (Table 1). The best enrichment score of those metabolic pathways in potato cultivars below N0 and N1 treatment indicated that multiple transcription differences strongly influenced the root, shoot, and leaf nitrogen metabolism.

Furthermore, the plant hormone auxin is critical for plant growth and development processes. It plays its regulatory role primarily by inducing the expression of early auxin response genes. The low nitrogen condition induced the biosynthesis of auxin and accumulation of transcripts, and the source of auxin or auxin transport revealed a role for auxin in regulating N remobilization [58, 59]. Using WGCNA and co-expression methodology, we identified many DEGs involved in auxin -induced and auxin-regulated (Figs. 4 and 6). We obtained several DEGs associated with the photosynthetic pathway and nitrogen metabolism pathway (Fig. 5). The eight DEGs *NR, NRT, NIR, NIRA, GS, GDH, CA*, and *formamidase* play important roles in methane metabolism, glyoxylate metabolism, and glutamate metabolism, which are associated with N metabolism. These DEGs and their functions are essential to potato NUE and trait variations [19, 20]. Finally, to identify the expression level and patterns, we selected fourteen DEGs from the 116, which were extremely co-expressed and connected with the traits. The *NRTs*, *auxin*-induced, *auxin*-regulated, *NRT*1.1, *chloroplast* pigment-binding, *chlorophyll* a-b binding, *ATP* synthase, wall-associated kinase, and *NADPH* genes kept consistent between RNA-Seq and qPCR detection. The candidate genes could be used for genetic manipulation for increasing NUE in potatoes via transgenic or CRISPR/Cas9 or base-editing technologies [44]. Exploring the molecular functions of these genes requires further experimental verification.

This study has several limitations: (i) This study did not conduct multiple growth stages rather one growth stage. (ii) The genetic correlation among multiple crop plants could not be conducted in this present study. (iii) The cultivar-specific expression pattern of genes and corresponding mutants’ performance throughout N deficiency were not presented in this present study. (iv) The newest potato genome DMv6.1, was not used in this present study which might be useful for distinguishing the isoforms. Therefore, we tend to shall use these in our future study of this subject with extended experiments.

## Conclusions

In conclusion, according to the RNA-Seq method and analyses, we obtain the whole-genome-wide transcriptional regulation and processes potentially implicated in response to N-deficiency in typical Qinghai potato plants. Metabolic pathways, like secondary metabolites including N-glycan, starch, lipid acid, amino acid, pigments, and vitamins; and nitrogen metabolism and photosynthesis caused multiple effects of nitrogen transporter and accumulation in the root, shoot and leaf under N-deficiency conditions. Hub-genes related to *NRTs*, *NRT*1.1, auxin induction, auxin regulation, chloroplast pigment binding, chlorophyll a-b binding, ATP synthase, wall-associated kinase, and NADPH lead to a biomass decrease in potato with N deficiency. The balance of plant hormones and N nutrients might regulate growth and development. The present study has greatly improved our knowledge of the enrichment of gene networks and regulatory elements involved in potato N metabolism pathways, strengthening future research on N metabolism and higher NUE in potatoes.

## Supplementary Information

**Additional file 1:  Table S1.** Primer sequences used for qRT-PCR.

**Additional file 2: Table S2.** RNA quality and quantity of 36 potato samples for RNA-Seq.

**Additional file 3: Table S3.** RNA-seq data summary and quality analysis.

**Additional file 4: Table S4.** The complete list of DEGs in different comparisons.

**Additional file 5: Table S5.** The genes overlapping in different comparisons in the Venn diagram.

**Additional file 6: Table S6.** The GO enrichment of DEGs and hub-genes in the root (R), shoot (S), and leaf (L) of N9 and 65 potatoes under N0 and N1 treatment.

**Additional file 7: Table S7.** List of 116 selected differentially expressed genes related to N transport and metabolism.

**Additional file 8: Figure S1.** Cluster diagram of all modules by WGCNA.

**Additional file 9: Figure S2.** The photosynthesis pathway and DEGs participated in the pathway.

## Data Availability

The datasets generated and/or analysed during the current study are available in the Bioproject accession: PRJNA741081 repository, https://www.ncbi.nlm.nih.gov/bioproject/PRJNA741081/.
