# Supplementary figures and images for: Transcriptome analysis reveals multiple effects of nitrogen accumulation and metabolism in the roots, shoots, and leaves of potato (Solanum tuberosum L.)
Source: BMC Plant Biol. 2022 Jun 9;22:282. doi: 10.1186/s12870-022-03652-3 (PMC9178895; doi:10.1186/s12870-022-03652-3)

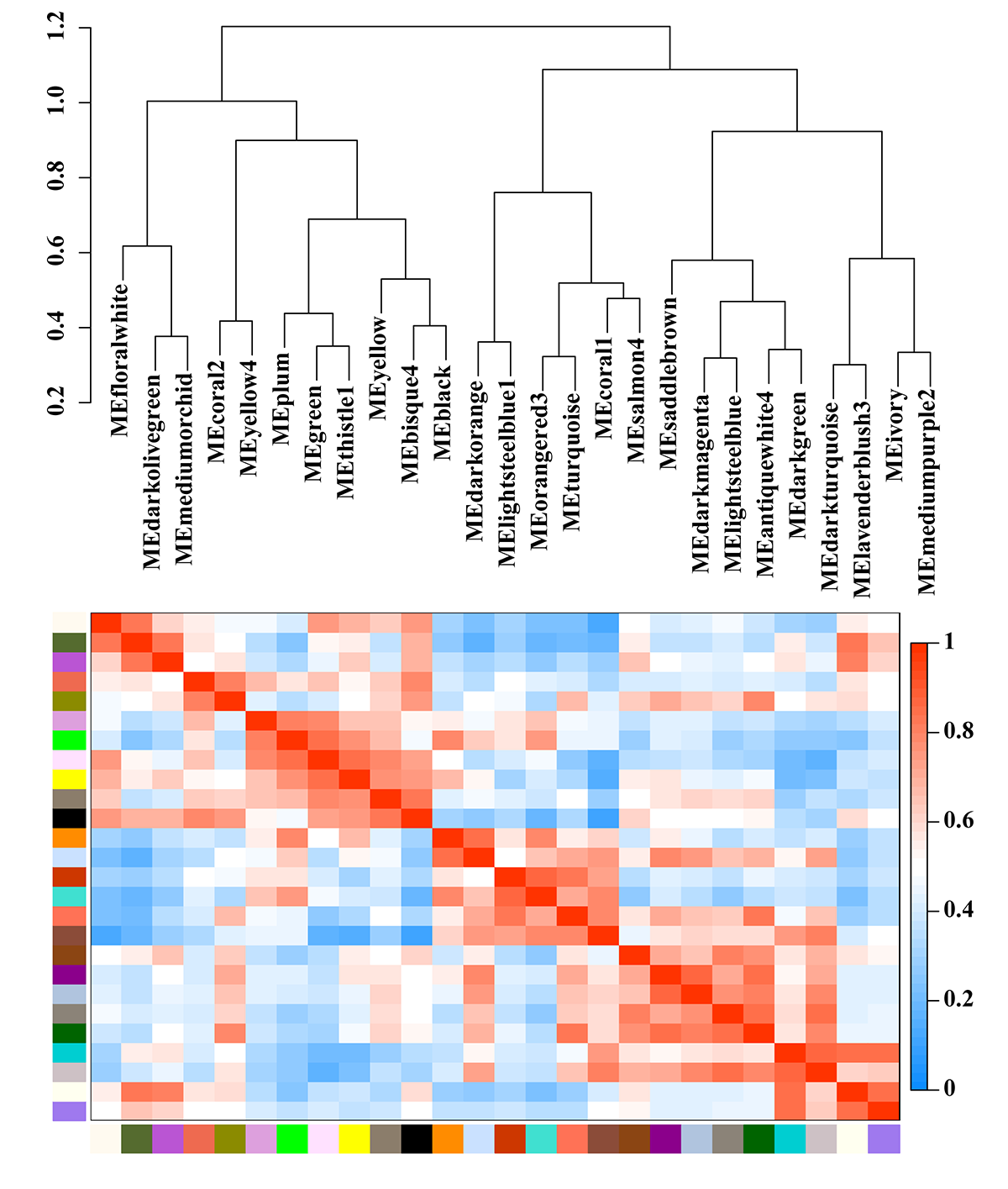

Supplement: Supplementary file 8 — Additional file 8: Figure S1. Cluster diagram of all modules by WGCNA. [file 12870_2022_3652_MOESM8_ESM.tif]

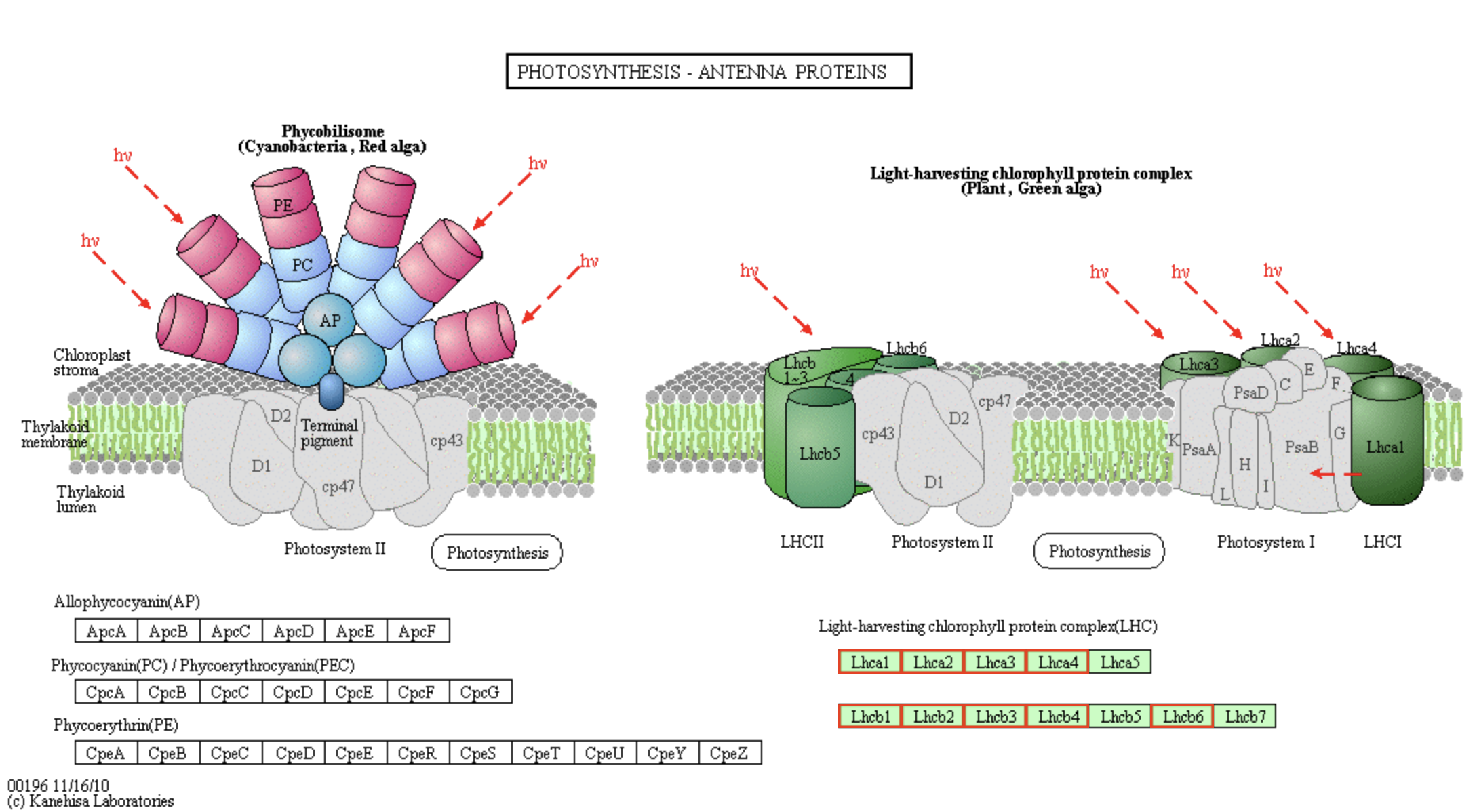

Supplement: Supplementary file 9 — Additional file 9: Figure S2. The photosynthesis pathway and DEGs participated in the pathway. [file 12870_2022_3652_MOESM9_ESM.tif]
